# Harnessing Microglia and Macrophages for the Treatment of Glioblastoma

**DOI:** 10.3389/fphar.2019.00506

**Published:** 2019-06-05

**Authors:** Ioanna Prionisti, Léo H. Bühler, Paul R. Walker, Renaud B. Jolivet

**Affiliations:** ^1^Division of Digestive and Transplantation Surgery, Geneva University Hospitals, Geneva, Switzerland; ^2^Lemanic Neuroscience Doctoral School, Geneva, Switzerland; ^3^Center for Translational Research in Onco-Hematology, Division of Oncology, Geneva University Hospitals – University of Geneva, Geneva, Switzerland; ^4^Département de Physique Nucléaire et Corpusculaire (DPNC), University of Geneva, Geneva, Switzerland; ^5^European Organization for Nuclear Research (CERN), Geneva, Switzerland

**Keywords:** macrophage, microglia, two-pore domain K^+^ channels, glioblastoma, cancer

## Abstract

Glioblastoma multiforme (GBM) is the most malignant form of brain tumors, with a dismal prognosis. During the course of the disease, microglia and macrophages both infiltrate the tumor microenvironment and contribute considerably in glioma development. Thus, tumor-associated microglia and macrophages have recently emerged as potentially key therapeutic targets. Here, we review the physiology of microglia and their responses in brain cancer. We further discuss current treatment options for GBM using radiotherapy, and novel advances in our knowledge of microglia physiology, with emphasis on the recently discovered pathway that controls the baseline motility of microglia processes. We argue that the latter pathway is an interesting therapeutic avenue to pursue for the treatment of glioblastoma.

## Introduction

As the resident immune cells of the Central Nervous System (CNS), microglia play key roles under both normal and pathological conditions. Microglia contribute to tissue homeostasis by actively surveying the brain, and by promoting the development of healthy neural networks by removing apoptotic cells, eliminating synapses and enhancing the production and survival of neuronal precursor cells (Wolf et al., 2017). However, when microglia are challenged, like for instance in the case of tumor formation, their immunological response can be strikingly suppressed, or maladapted (Kettenmann et al., 2011). Glioblastoma multiforme (GBM) is the most malignant form of primary brain tumor, characterized by significant infiltration of resident microglia and peripheral macrophages in the tumor, and by pervasive infiltration of tumor cells in the healthy surroundings of the tumor. Advances in our understanding of microglial physiology and in our understanding of the complex interactions between microglia and tumor cells in GBM can elucidate their role in glioma progression and indicate potentially interesting druggable targets. Here, we argue that the two-pore domain potassium channel THIK-1 (Tandem-pore domain Halothane-Inhibited K^+^ channel; *Knck13*) might be such a target. THIK-1 was recently identified by some of us as a key “hub” mechanism regulating microglia ramification, baseline motility of processes and release of interleukin-1β (Madry et al., 2018; Kyrargyri et al., unpublished). In the following, we briefly review key aspects of microglia physiology before moving on to tumor-associated microglia and macrophages (TAMs). We then conclude by reviewing current and future treatment options for GBM, highlighting how targeting THIK-1 could be harnessed to complement these.

## Physiology of Microglia

Microglia are the resident mononuclear macrophages of the CNS, and constitute ∼5–20% of all glial cells in the CNS parenchyma (Saijo and Glass, 2011; Ginhoux et al., 2013; Sousa et al., 2017). These cells are heterogeneously distributed in non-overlapping regions throughout the brain and spinal cord (Lawson et al., 1990). In addition to parenchymal microglia, the CNS macrophage population includes non-parenchymal perivascular, meningeal and choroid plexus border-associated macrophages (BAMs) (Ransohoff and Cardona, 2010; Saijo and Glass, 2011; Goldmann et al., 2016; Mrdjen et al., 2018). The origin of microglia has been controversial since Pío del Río Hortega first introduced the concept of microglia and argued that these cells are of mesodermal origin and enter the brain during early development (Sierra et al., 2016). Recent studies have shown that microglia originate from primitive hematopoiesis in the fetal yolk sac and populate the brain during embryonic development (Ginhoux et al., 2010; Saijo and Glass, 2011; Prinz and Priller, 2014). Although microglia and brain macrophages were considered two ontogenetically distinct populations, new fate-mapping approaches have challenged this assumption (Prinz and Priller, 2014; Goldmann et al., 2016; Greter, 2016).

During development and in the adult brain, microglial cells play an important role, extensively interacting with neuronal circuits (Squarzoni et al., 2014; Miyamoto et al., 2016). They accommodate neuronal apoptosis, eliminate less active synaptic connections (synaptic pruning) and regulate neuronal activity (Paolicelli et al., 2011; Li et al., 2012). Interestingly, recent reports have indicated that microglia also promote synapse formation in the mature brain (Parkhurst et al., 2013; Miyamoto et al., 2016).

In the healthy adult CNS, microglia exhibit a not very aptly named “resting” phenotype, characterized by small cellular bodies from which thin ramified processes are extended. *In vivo* two-photon imaging studies have, however, demonstrated that these protrusions are highly motile, providing a kind of continuous surveillance of the extracellular space (Davalos et al., 2005; Nimmerjahn et al., 2005). Thus, microglia in their so-called “resting” state are not dormant, but instead actively scan their environment, ready to respond upon a threat on the CNS (Davalos et al., 2005; Kettenmann et al., 2011). The transition from the “resting” to the “activated” state under pathological conditions, such as inflammation or disease, implies not only functional but also morphological alterations. Highly “activated” microglia can take up an amoeboid shape, becoming morphologically indistinguishable from other macrophages (Boche et al., 2013). Depending on their state, microglia exhibit different types of motility (Kettenmann et al., 2011). Resting microglia survey the brain by constantly extending and retracting their ramified processes, without translocation of the cellular body (Nimmerjahn et al., 2005). Some of us have recently demonstrated that this baseline surveillance of the parenchyma by microglia is controlled by the two-pore domain potassium channel THIK-1 (see below) (Madry et al., 2018). The convergence of microglial processes toward a damaged area is triggered by “danger signals,” like extracellular ATP and its derivatives, which target purinoreceptors of the P2Y family (Davalos et al., 2005; Burnstock and Verkhratsky, 2010). In particular, P2Y_12_ receptors are highly expressed in resting microglia, but they are reduced after microglial activation (Haynes et al., 2006). The link between process outgrowth and ATP release is further reinforced by the observation that P2Y_12_ proteins aggregate at the bulbous tips formed at the end of microglial branches upon ATP stimulation (Dissing-Olesen et al., 2014). In contrast with this baseline surveillance operated by resting, ramified, microglia, under pathological conditions, amoeboid microglia move in their entirety while migrating to the site of injury (Wolf et al., 2017).

In the diseased CNS, the blood brain barrier (BBB) is usually impaired, leading to an infiltration of peripheral macrophages (Hambardzumyan et al., 2016). Under tissue damage, macrophages can express two types of activation; the classical activation (M1) is a pro-inflammatory state, while the alternative activation (M2) is defined as the anti-inflammatory state. However, the concept of M1/M2 polarization is considered oversimplified in the case of microglia (Nakagawa and Chiba, 2014; Gabrusiewicz et al., 2016; Ransohoff, 2016; Broekman et al., 2018), with no clear dichotomy reported in GBM (Gabrusiewicz et al., 2016). Resident microglia express pattern recognition receptors (PRRs), which detect pathogen-associated molecular patterns (PAMPs), such as microbial pathogens, and damage-associated molecular patterns (DAMPs), like adenine nucleotides (ATP/ADP). PAMPs and DAMPs are though counteracted by glycans known as self-associated molecular patterns (SAMPs), which appear modified in tumor cells, inhibiting immune response in their surroundings (Rodriguez et al., 2018). The exploitation of glycans by cancer cells promotes immune suppression by controlling the differentiation of TAMs (Rodriguez et al., 2018). During an infection, the microglial immune response is mediated via several pathways, including transmembrane proteins known as Toll-like receptors (TLRs) (Lehnardt, 2010) and the cytoplasmic NOD-like receptors (NLRs). NLRP3 is a subset of the NLR family that, together with the adaptor protein ASC and Caspase-1, form the NLRP3 inflammasome (Walsh et al., 2014; Wolf et al., 2017). Activation of NLRP3, followed by activation of Caspase-1, results in the production and release of interleukin-1β (IL-1β) and interleukin-18 (IL-18) (Walsh et al., 2014; Wolf et al., 2017). High expression levels of NLRP3 in microglia (Zhang et al., 2014) and the contribution of IL-1β in the development and progression of malignant tumors (Voronov et al., 2003; Yuzhalin and Kutikhin, 2015) create new interesting directions for future cancer studies. Again, some of us have shown that NLRP3 activation and subsequent IL-1β release by microglia is also controlled by the same THIK-1-related pathway that controls baseline surveillance of the parenchyma by microglia (Madry et al., 2018).

A clear distinction between activated microglia and infiltrating macrophages is impeded due to their common myeloid lineage (Kettenmann et al., 2011). Nonetheless, several markers have been identified and are currently used to distinguish these two populations in the CNS. The ionized calcium-binding adaptor molecule 1 (Iba1) and the human fructose transporter 5 (GLUT5) are suggested as useful markers for both resting and activated microglia (Ito et al., 1998; Horikoshi et al., 2003; Sasaki, 2016). Microglia, which are able to generate ATP by both glycolysis and oxidative phosphorylation, highly express GLUT5, which has a high affinity for fructose (Ghosh et al., 2018). However, since the brain shows low concentrations of fructose, the function of GLUT5 in microglia, and this is true in general for vast swaths of their metabolism, remains uncertain (Payne et al., 1997; Douard and Ferraris, 2008; Caruso et al., 2018). Moreover, the use of CD45 antibodies has shown low expression levels for resident microglia (CD45^low^) and high expression levels for CNS macrophages (CD45^high^) (Kettenmann et al., 2011), while CD49D was absent in microglia and can be used to distinguish them from CNS macrophages in mouse and human brain tumors (Bowman et al., 2016). Other microglia markers include the major histocompatibility complex (MHC) class II, the fractalkine receptor (CX_3_CR1), and the recently identified Sall1, which can be used to discriminate parenchymal microglia from BAMs (Davalos et al., 2005; Buttgereit et al., 2016; Sasaki, 2016; Mrdjen et al., 2018).

Transcriptome analysis provides the tools to discriminate microglia not only from the peripheral macrophages, but also among the other cell populations of the nervous system (Gautier et al., 2012; Lavin et al., 2014; Zhang et al., 2014). Neurons, macroglia (astrocytes, oligodendrocytes) and vascular cells express no morphological resemblance with resting or activated microglia. However, gene expression profiles and immunophenotyping can provide insights into the functions of the different cell types of the CNS under normal and pathological conditions (Ginhoux et al., 2010; Gautier et al., 2012; Lavin et al., 2014; Zhang et al., 2014; Wieghofer et al., 2015). In particular, these genetic tools combined with imaging techniques can interpret the role of microglia and infiltrating macrophages in a number of diseases, such as brain cancer.

## Tumor-Associated Microglia and Macrophages in Glioblastoma

**FIGURE 1:**
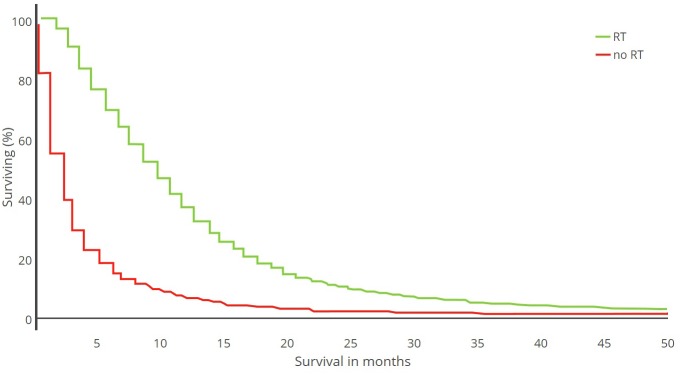
Kaplan–Meier plot. Survival analysis of 21,783 GBM patients treated with radiotherapy (RT) versus no RT (1973–2007) [adapted from Zinn et al. (2013)].

While both resident microglia and macrophages are the main innate immune cells of the CNS, their role may be subverted in case of certain pathological insults. In brain cancer, these macrophage populations infiltrate the tumor area and can contribute to up to 50% of non-neoplastic cells, raising the possibility for new therapeutic strategies (Hambardzumyan et al., 2016). GBM (World Health Organization grade IV astrocytoma) is the most common type of malignant primary brain tumor, carrying a poor prognosis and high rate of recurrence (Louis et al., 2007) (Figure 1). It also contains cancer stem cells (CSCs), which contribute to tumor initiation and therapeutic resistance (Lathia et al., 2015). According to gene expression profiling based on data from The Cancer Genome Atlas (TCGA)^1^, there are three molecular subtypes of GBM defined, including proneural, classical and mesenchymal (Verhaak et al., 2010; Sidaway, 2017). These subtypes are characterized by the patterns of alterations of the *EGFR*, *NF1*, *PDGFRA* and *IDH1* genes, in addition to their response to therapy (Crespo et al., 2015; Verhaak et al., 2010). However, phenotypic shifts are occurring upon treatment and relapse (Garnier et al., 2018). The implication of TAMs in several aspects of glioma development, including proliferation, angiogenesis and immunosuppression, contributes to the therapeutic resistance and the short survival rate of this malignant tumor (Schonberg et al., 2013; Hambardzumyan et al., 2016). Moreover, the gene-expression GBM subtype may be directly linked to microglia and TAM infiltration, with the mesenchymal subtype being associated with high infiltration, and even being driven by the presence of these infiltrating cells (Bhat et al., 2013; Wang et al., 2017). GBM is subdivided into primary GBM, which arises *de novo* without prior low-grade disease, and secondary GBM, deriving from previously detected low grade astrocytomas (Furnari et al., 2007). Genetic analysis of human GBM has shown frequent and diverse alterations in the *IDH1* gene among others, which lead to a reclassification of GBM as IDH-mutant or IDH-wild type, with IDH-mutant having better prognosis (Chin et al., 2008; Parsons et al., 2008; McFaline-Figueroa and Lee, 2018). IDH-mutant is further subdivided into two major types of glioma: astrocytoma (IDH-A) and oligodendroglioma (IDH-O), which differ genetically and histopathologically as shown by single-cell RNA sequencing analysis (Venteicher et al., 2017). Supplementary studies on genetic aberrations in GBM could provide more reliable diagnostic tools and patient-specific targeted therapies.

**FIGURE 2:**
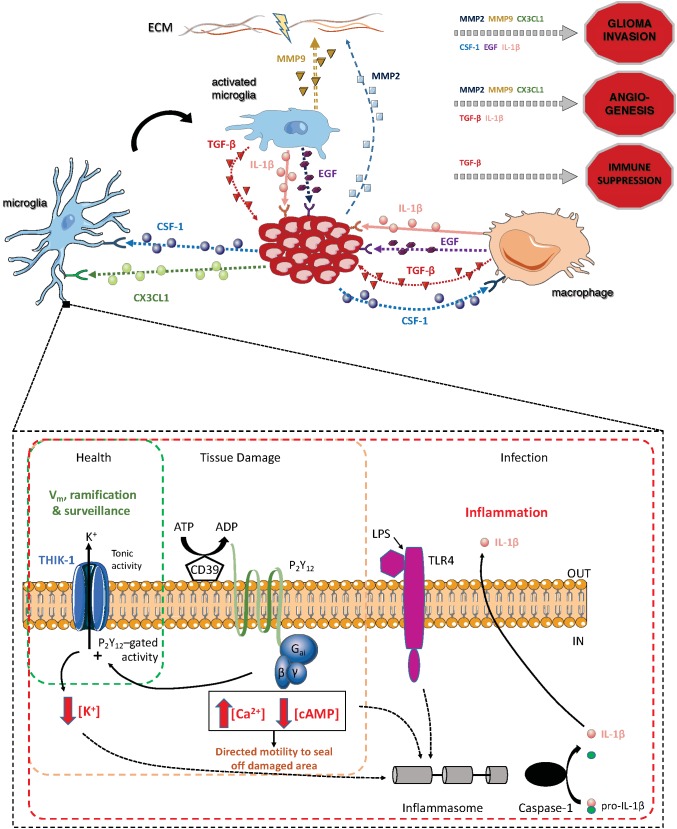
Summary of the main pathways through which TAMs and glioma cells interact, and summary of the main pathways in microglia in which the two-pore domain potassium channel THIK-1 is involved [that second part adapted from Madry et al. (2018)].

The recruitment of microglia and peripheral macrophages in the surroundings of the tumor is controlled via the release of several chemoattractants, including fractalkine (CX3CL1) whose receptor, CX3CR1, is mostly expressed by microglia in adults (Hambardzumyan et al., 2016). Chemoattraction by osteopontin was also recently reported in GBM, binding to macrophage-expressed integrin αvβ5 (Wei et al., 2019). Upon accumulation to the glioma site, the functions of both microglia and macrophages are subverted and they can amplify tumor-mediated immunosuppression and promote tumor invasiveness (Reardon et al., 2017) (Figure 2). The expression of matrix metalloproteinases (MMPs), which degrade the extracellular matrix in the glioma microenvironment is associated with higher glioma invasion and angiogenesis (Poon et al., 2017). Notably, the activation of the CX3CL1/CX3CR1 system has been indicated to upregulate the expression of the gelatinases (MMP2, MMP9) and the membrane- associated MT1-MMP (or MMP14) (Held-Feindt et al., 2010). Further studies have highlighted the increased expression of MMP9 and MT1-MMP in TAMs via TLR2 signaling and p38 mitogen-activated protein kinase (MAPK) pathway (Charles et al., 2011; Vinnakota et al., 2013; Hu et al., 2014). TAMs release several anti-inflammatory factors such as transforming growth factor beta (TGF-β) and vascular endothelial growth factor (VEGF), that promote immune suppression and tumor angiogenesis (Watters et al., 2005). Results derived from the study of glioma stem-like cells (GSLCs) indicated that their invasiveness is enhanced following the release of TGF-β1 from TAMs, which increases MMP9 expression (Ye et al., 2012). Microglial MMP9 is suggested to promote glioma motility and enhance angiogenesis via VEGF regulation (Lee et al., 2005; Coniglio and Segall, 2013; Hu et al., 2014). Epidermal growth factor (EGF) and colony-stimulating factor-1 (CSF-1) have also been implicated in TAM glioma crosstalk. The microglial-released EGF increases tumor invasion by activating its receptors on GBM cells, while CSF-1 secreted by glioma acts as a chemoattractant for TAMs (Coniglio et al., 2012). Indeed, it was shown that CSF-1R inhibition alters macrophage polarization and blocks proneural glioma progression (Pyonteck et al., 2013; Quail et al., 2016). IL-1β is an isoform of the IL-1 cytokine superfamily secreted mainly by immune cells, including TAMs, while its receptor (IL-1R) has been found in glioma cells (Sasaki et al., 1998). Several studies have demonstrated that IL-1β is able to activate both MMP9 and VEGF, thus stimulating tumor invasiveness and angiogenesis (Sasaki et al., 1998; Voronov et al., 2003; Yuzhalin and Kutikhin, 2015). Moreover, IL-1β production has been shown to increase the expression of other cytokines in glioma microenvironment, such as IL-6 and IL-8, which also have crucial role in tumor development (Yeung et al., 2013). Microglial release of IL-1β can also be driven by the neuropeptide substance P (SP), which is expressed in both microglia and glioma cells, along with its receptor NK-1 (Rasley et al., 2002; Watters et al., 2005; Cordier et al., 2014). In the brain, microglia are the main source of IL-1β and the main K^+^ channel they express at rest (THIK-1) plays an important role in IL-1β production (Madry et al., 2018), suggesting a potential role for this channel in GBM progression.

## Radiotherapy and New Approaches in Glioblastoma Treatment

The standard treatment for GBM is surgery, followed by adjuvant radiotherapy and chemotherapy. However, none of these treatments, alone or in combination, are effective enough to increase the median survival of 15 months. The use of ionizing radiation encounters considerable challenges in cancer treatment by the need to deliver sufficient energy to the tumor area without damaging the surrounding healthy tissue (Zhao et al., 2016). Furthermore, the antitumor effects of radiotherapy in tumor microenvironment are still controversial (Vatner and Formenti, 2015). It has been demonstrated that the recruitment of TAMs in glioma site is enhanced in response to radiotherapy (Vatner and Formenti, 2015). There is also a correlation between the presence of M2 glioma infiltrating macrophages and radiotherapy resistance (Wang et al., 2018). Glioma stem cells (GSCs) under temozolomide (TMZ) chemoradiation have been found to evolve from a TMZ-sensitive to a TMZ-resistant state (Garnier et al., 2018). Studies in prostate cancer indicated that the recruitment of myeloid cells results from the higher expression of CSF-1 in tumor cells following radiation (Xu et al., 2013). In addition, high doses of ionizing radiation upregulate the pro-inflammatory cytokines IL-1β and IL-6 (Betlazar et al., 2016). Despite the challenges, radiation therapy continues to be used for the treatment of malignant tumors. The efficacy of the treatment has been significantly enhanced with the development of image-guided techniques and the use of nanoparticle carriers as theranostic agents (Phillips et al., 2014).

In radiation therapy - external, internal and systemic - tumor cells are bombarded with ionizing radiation, such as α- and β-emitting radionuclides, X-rays, γ rays and Auger emitters. External beam radiotherapy (EBRT) has been the benchmark for radiation therapy for the last decades, delivering high energy X-rays from outside the body (Zhao et al., 2016). In stereotactic radiosurgery (SRS), a focused high radiation dose targets a well-defined tumor area, hence minimizing the effect of radiation in healthy tissue (Barani and Larson, 2015). Hadrontherapy is a form of radiotherapy that uses charged particles, such as protons and other ions, to irradiate the tumor. The use of particles instead of X-rays allows the precise definition of the tumor area, while minimizing the damage to the surrounding healthy tissue (Orecchia et al., 1998). In systemic radiation therapy, the radioactive sources are either ingested, infused or intravenously delivered (Zhang et al., 2010). Brachytherapy is a form of internal radiation where radioisotopes are placed inside or next to the tumor via craniotomy or stereotactic techniques (Vitaz et al., 2005). Image-guidance, such as positron emission tomography (PET) and single photon emission computed tomography (SPECT), is often used in combination with brachytherapy to monitor the tumor area and direct the nanoparticles accordingly (Phillips et al., 2014).

Nanoparticles have been a significant boost in glioma diagnosis and treatment (Glaser et al., 2017). The ability of nanoparticles to be conjugated with biological molecules or other receptor ligands potentiate their affinity to the tumor microenvironment and the delivery of tumor-targeted radioisotopes. Since the cellular expression between normal and tumor cells differs, the engineering of nanoparticles with molecules that target highly expressed tumor receptors is a promising area of nanomedicine research (Hernandez-Pedro et al., 2013). Both glioma cells and TAMs have become targets of radionuclide carriers but without any promising outcome so far. On the other hand, labeling nanoparticles with substance P (SP) antagonist is a promising method, though it will require further investigations (Cordier et al., 2014). Further research is also needed to determine the appropriate radioisotopes for each tumor type radiotherapy, also taking into account the chemical and physical properties of the selected isotope. It has been speculated that the recruitment of TAMs by tumor cells could be a potential approach for drug delivery (Phillips et al., 2014; Poon et al., 2017).

Immunotherapies and immunotherapy combinations have lately emerged as having the potential to offer benefit in brain cancer (Dutoit et al., 2016; Reardon et al., 2017). There are also 4 types of gene therapy currently being studied for GBM treatment by targeting the tumor area with minimum effects in the surrounding healthy tissue; suicide genes, immunomodulatory genes, tumor-suppressor genes and oncolytic virotherapy (Kane et al., 2015). Oncolytic viruses (OVs) can be engineered for tumor cell specificity and injected directly into the glioma site during surgery (Lawler et al., 2017). Several clinical studies for GBM and recurrent GBM tested oncolytic viruses, such as adenovirus and polio virus, as cancer therapeutics, but the challenge is to avoid early clearance of the OVs from the patient’s immune system (Desjardins et al., 2016; Lawler et al., 2017). Nevertheless, the field is advancing rapidly, with macrophages considered to be a critical element that can dictate resistance or responsiveness to virotherapy (Saha et al., 2017; Delwar et al., 2018).

Another medical approach that is gaining increasing consideration is drug repositioning, which is defined as the investigation and use of already approved drugs for different therapeutic indications, including cancer. In this direction, several drugs are proposed for GBM treatment in combination with temozolomide (TMZ), such as *disulfiram*, which can cross the blood-brain barrier (BBB) and *metformin*, which has been shown to inhibit CSCs proliferation (Gritti et al., 2014; Wurth et al., 2016). A further approach has employed tricyclic antidepressants with the anticoagulant ticlopidine to induce cell-lethal autophagy in human and mouse glioma cells, and in mouse models *in vivo* (Shchors et al., 2015). Moreover, a new method for recurrent GBM called “coordinated undermining of survival paths with nine repurposed drugs,” or CUSP9^∗^, has been suggested in combination with TMZ (Kast et al., 2014).

In the context highlighted above, we argue that targeting the two-pore domain channel THIK-1 in microglia is an interesting therapeutic route to follow. THIK-1 was recently demonstrated to be the main K^+^ channel in resting microglia, tonically active regardless of the state of P2Y_12_ receptors, and the largest contributor to microglia’s resting membrane potential (Madry et al., 2018). We have also established that THIK-1 activity determines microglial ramification, surveillance and is involved in IL-1β release. The contribution of IL-1β, whose receptors are expressed by glioma cells, in the development and progression of GBM has been extensively documented (Voronov et al., 2003; Feng et al., 2015). Additionally, targeting THIK-1 will impact microglia motility and the cellular machinery that supports it, which is intricately linked to the structure of the extracellular matrix, while it has been shown that diverse soluble factors released by glioma cells promote the degradation of the extracellular matrix by microglia, increasing the invasiveness of glioma cells. We believe that the fact that THIK-1 controls both IL-1β production and cellular motility in microglia makes this channel a very interesting target for the treatment of GBM, with the potential to impact both the tumor growth and its invasiveness. Microglia express two distinct motility modes, but only microglial ramification and surveillance depend on the tonic activity of THIK-1 (the convergence of microglial processes toward a damaged area is independent from THIK-1). Thus, THIK-1 inhibition could repress the capability of resting microglia close to glioma site to expand their processes during surveillance, preventing them from being recruited by cancer cells, but it would also limit IL-1β production, which, as we discussed above, is involved in glioma progression. Given that GBM growth relies on TAM recruitment, THIK-1 blockade could severely limit microglial involvement. Unfortunately, it is for now very difficult to target THIK-1 pharmacologically as this channel has not yet been extensively studied. Preclinical trials can of course be performed in THIK-1 knockout animals. SiRNA therapeutics have been studied in cancer clinical trials (Shen et al., 2012) and could be a potential approach for the treatment of glioblastoma in that context. The use of nanoparticles can be a means to direct these gene modulators in inhibiting THIK-1 (Es-Salah-Lamoureux et al., 2010). Whether the removal of this specific channel will also have negative consequences for different cell functions remains an open question. Interestingly, blocking other members of the two-pore-domain potassium channel family, like TASK-3 or TREK-1, has shown significant reduction in cell proliferation in some ovarian cancer cell lines (Comes et al., 2015), which appears to be a promising path for other cancer cells, like gliomas.

## Conclusion

Since the advent of the current standard therapy for newly diagnosed GBM consisting of surgical resection and chemoradiotherapy, there have been no major treatment advances, with the possible exception of mitosis-disrupting tumor-treating fields (TTFields) (Geraldo et al., 2019). New insights into the inter- and intra-tumoral genetic heterogeneity of GBM (Patel et al., 2014; Sidaway, 2017) highlight the likely futility of discovering tumor cell-targeted therapies with therapeutic impact on sufficient patients, or on sufficient tumor cells within the tumor mass. Nevertheless, we argue that there is one common feature of GBM that can be potentially targeted: the massive infiltrate of microglia and macrophages. Non-mutated, widely expressed microglial cell targets such as the THIK-1 K^+^ channel offer an opportunity to modulate the GBM stroma and to potentially tilt the balance of the multiple factors in the tumor microenvironment away from tumor promotion (Broekman et al., 2018). The future for treating highly aggressive, heterogenous, and therapy resistant malignancies such as GBM will likely be a rational combination of different therapeutic modalities. Critically, microglia and macrophages have been shown to influence efficacy (positively or negatively) of many treatments including chemotherapy, radiotherapy, virotherapy and immunotherapy. Consequently, we envisage that harnessing these cells for therapeutic advantage will be at the center of future, more potent, GBM therapies.

## Author Contributions

All authors listed have made a substantial, direct and intellectual contribution to the work, and approved it for publication.

## Conflict of Interest Statement

The authors declare that the research was conducted in the absence of any commercial or financial relationships that could be construed as a potential conflict of interest.

## Footnotes

1 http://cancergenome.nih.gov/
